# Two new records of palm species for Gabon: *Sclerosperma profizianum* Valk. & Sunder. and *Eremospatha quiquecostulata* Becc.

**DOI:** 10.3897/BDJ.4.e10187

**Published:** 2016-11-18

**Authors:** Paul Henri Bourobou Bourobou, Raoul Niangadouma, Yves Issembe, Thomas L.P. Couvreur

**Affiliations:** ‡ Institut de Pharmacopée et Médecines Traditionnelles (IPHAMETRA), Centre National de la Recherche Scientifique et Technique (CENAREST), Libreville, Gabon; § Herbier National du Gabon, Libreville, Gabon; | Institut de Recherche pour le Développement (IRD), Montpellier, France; ¶ Université de Yaoundé I, Ecole Normale Supérieure, Département des Sciences Biologiques, Yaoundé, Cameroon

**Keywords:** Arecaceae, rattan, flora, Gabon, new records

## Abstract

**Background:**

Gabon is an important center of biodiversity in Central Africa. The country contains to date 27 species of palms. However, palms are generally poorly collected as these massive plants are hard to press and curate. Thus, our understanding remains incomplete, especially in Central Africa.

**New information:**

We report three new records of two palm species for Gabon: *Sclerosperma profizianum* Valk. & Sunder. and *Eremospatha quiquecostulata* Becc.. The former species was collected in southeast Gabon, near Ndindi, while the later was collected in the Massif du Chaillu and Monts de Cristal National Park (Crystal Mountains National Park). The total number of palm species for Gabon is now 29, making it an important centre of palm diversity in Central Africa.

## Introduction

Although only 270.000 km² in size, the Central African country of Gabon is an important center of botanical biodiversity for the continent. Indeed, the check-list of vascular plant species published by Sosef et al. (2006) indicates that Gabon contains around 4710 species. In addition, the soon to be completed Flore du Gabon volumes suggest we have a relatively well known botanical knowledge of Gabon (Sosef, pers. com.). Yet, new discoveries are regularly published such as new genera (Couvreur et al. 2015, Wieringa et al. 2013) new species (Hoekstra et al. 2014, Couvreur and Niangadouma 2016, Lachenaud and Breteler 2011, van Velzen and Wieringa 2014, Wieringa et al. 2013) and signalizations of new species for the country are common.

Palms are not very diverse in Africa with just 65 species recorded to date, in stark contrast to South America (800 species) and South East Asia (1200 species) (Blach-Overgaard et al. 2013, Dransfield et al. 2008). To date Gabon contains eleven genera and 27 species of palms (van Valkenburg 2006). Several important works exist on African palms (in Central Africa: Letouzey 1978, Stauffer et al. 2014, Sunderland 2007, Sunderland 2012, Tuley 1995, van Valkenburg and Sunderland 2008, van Valkenburg et al. 2008). Yet, our knowledge of African palms remains incomplete mainly because they are rarely/partially collected. However, new species are still regularly published (Couvreur and Niangadouma 2016, Sunderland 2002, Sunderland 2003, van Valkenburg et al. 2008) and new species are awaiting description (Faye et al. 2016).

Here, we record two new palm species for Gabon and provide updated distribution maps for both species as well as a key to the species of *Eremospatha* of Gabon.

## Materials and methods

Two field trips were undertaken. The first in February 2015 to the area around Ndindi (region Nyanga) commissioned by the petrol company Maurel and Prom for environmental impact studies. The second in June 2016 to the Massif du Chaillu (area around the town of Koulamotou, Ogooué-Lolo) and Crystal Mountains National Park, Mbé Sector (around Kinguélé and Tchimabélé, Estuaire & Woleu-Ntem regions). This latter trip was undertaken as part of the ARFODYN and RAPHIA projects.

Traditional herbarium collection methods for palms were employed (Dransfield 1986). Specimens were collected in the field and dried. An original set for each collection was deposited at the Herbier National du Gabon in Libreville (LBV). Duplicates were then sent out to other herbaria (G, K, P, WAG).

Identification of specimens was done at the Herbier National du Gabon and using literature (Sosef et al. 2006, Sunderland 2012, Sunderland 2007, van Valkenburg et al. 2008). We also used the recently generated RAINBIO mega database which provides distribution data for over 24.000 species across tropical Africa based on a compilation of over 600.000 georeferenced herbarium specimens (Dauby et al. 2016). RAINBIO contains over 90% of all botanical collections made in Gabon (Sosef et al. 2006). Distribution maps were made using QGIS Lyon version.

## Taxon treatments

### Eremospatha quiquecostulata

Becc. 1910

urn:lsid:ipni.org:names:666847-1

**Figure 1.**
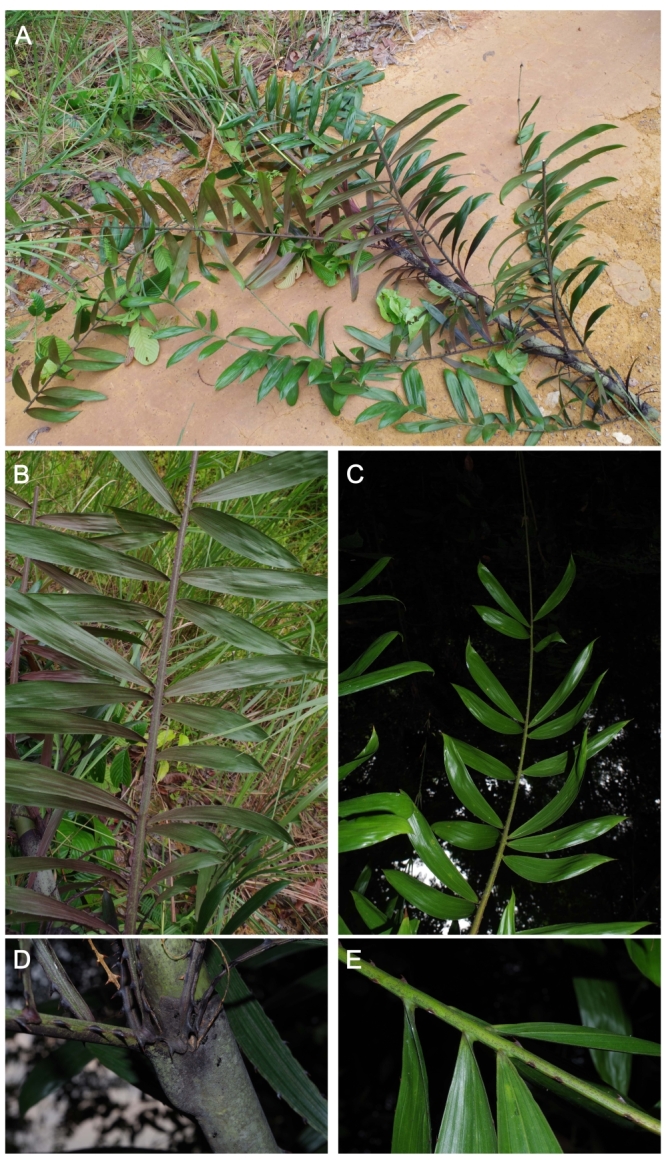
*Eremospatha quiquecostulata*. A) Detail of collection Couvreur 1079; B) Detail of inequidistant leaflets, characteristic of the species; C) View of the leaf and the flagellum; D) Detail of the leaf base showing the knee; E) Detail of the grouping of the leaflets in 2-3. Photos TLP Couvreur.

Arecaceae
Eremospatha quiquecostulata Becc. Webbia iii. 279. 1910 (Beccari 1910). Type: Cameroon, Dja, unknown collector (FI). (Fig. 1)

#### Materials

- **Type status:** Other material. **Occurrence:** recordNumber: T.L.P. Couvreur 1079; recordedBy: Thomas Couvreur; **Taxon:** taxonID: urn:lsid:ipni.org:names:666847-1; scientificName: Eremospatha quiquecostulata; kingdom: Plantae; class: Magnoliopsida ; order: Arecales; family: Arecaceae; genus: Eremospatha; specificEpithet: quiquecostulata; scientificNameAuthorship: Becc.; **Location:** continent: Africa; country: Gabon; stateProvince: Estuaire; locality: Monts de Cristal National Park, Mbé sector, 800 m from Kinguélé ANPN camp, near bridge; locationRemarks: label transliteration: "Gabon, Ogooué-Lolo, Road Koulamotou - Pana, km 45, after Lemjene village. S 1.45585, E 12.5863]"; verbatimCoordinates: 1° 27' 21.06'' S; 12° 35' 10.68'' E; verbatimLatitude: -1° 27' 21.06"; verbatimLongitude: 12° 35' 10.68''; decimalLatitude: -1.45585; decimalLongitude: 12.5900; geodeticDatum: WGS84; georeferenceProtocol: GPS; **Identification:** identifiedBy: Thoms L.P. Couvreur; dateIdentified: 2016; **Event:** eventDate: 2016-06-03; year: 2016; month: 6; day: 3; **Record Level:** language: english; collectionID: urn:lsid:biocol.org:col:34252; collectionCode: WAG; basisOfRecord: PreservedSpecimen
- **Type status:** Other material. **Occurrence:** recordNumber: T.L.P. Couvreur 1144; recordedBy: Thomas Couvreur; **Taxon:** taxonID: urn:lsid:ipni.org:names:666847-1; scientificName: Eremospatha quiquecostulata; kingdom: Plantae; class: Magnoliopsida ; order: Arecales; family: Arecaceae; genus: Eremospatha; specificEpithet: quiquecostulata; scientificNameAuthorship: Becc.; **Location:** continent: Africa; country: Gabon; stateProvince: Ogooué-Lolo; locality: Road Koulamotou - Pana, km 45, after Lemjene village; locationRemarks: label transliteration: "Gabon, Estuaire, Monts de Cristal National Park, Mbé sector, 800 m from Kinguélé ANPN camp, near bridge. N 0.46394, E 10.27856]"; verbatimCoordinates: 0° 27' 50.184'' N; 10° 16' 42.816'' E; verbatimLatitude: 0° 27' 50.184''; verbatimLongitude: 10° 16' 42.816''; decimalLatitude: 0.46; decimalLongitude: 10.27856; geodeticDatum: WGS84; georeferenceProtocol: GPS; **Identification:** identifiedBy: Thoms L.P. Couvreur; dateIdentified: 2016; **Event:** eventDate: 2016-06-14; year: 2016; month: 6; day: 14; **Record Level:** language: english; collectionID: urn:lsid:biocol.org:col:34252; collectionCode: WAG; basisOfRecord: PreservedSpecimen

#### Distribution

**Figure 2.**
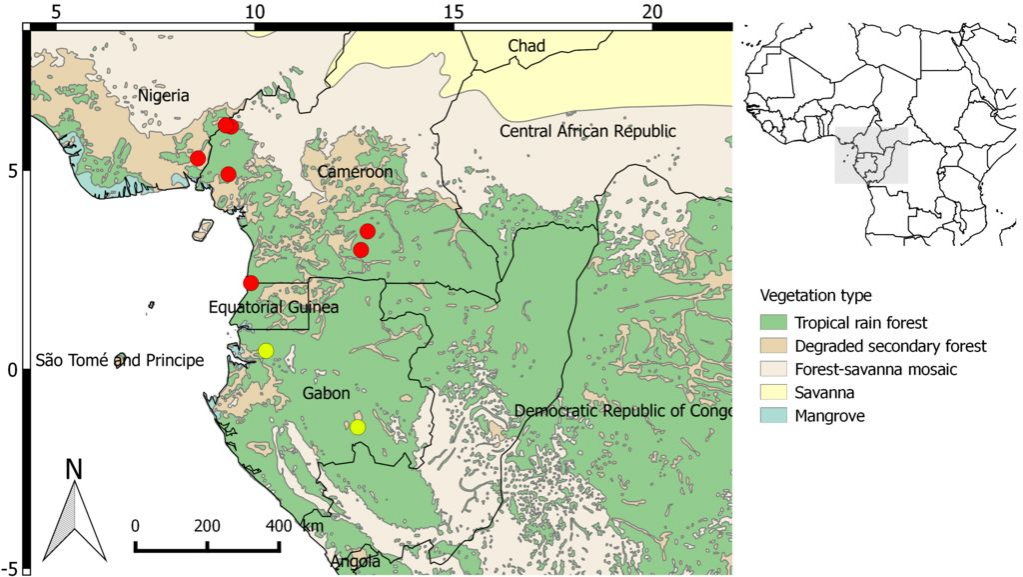
Updated distribution map for *Eremospatha quiquecostulata*. Yellow dots: New collections published here; Red dots: distribution points from the RAINBIO dataset.

Documented occurrences: Cameroon, with a single collection in Nigeria and one in Equatorial Guinea Sunderland 2012 (Fig. 2).

### Sclerosperma profizianum

Valk. & Sunderl. 2008

urn:lsid:ipni.org:names:77090324-1

**Figure 3.**
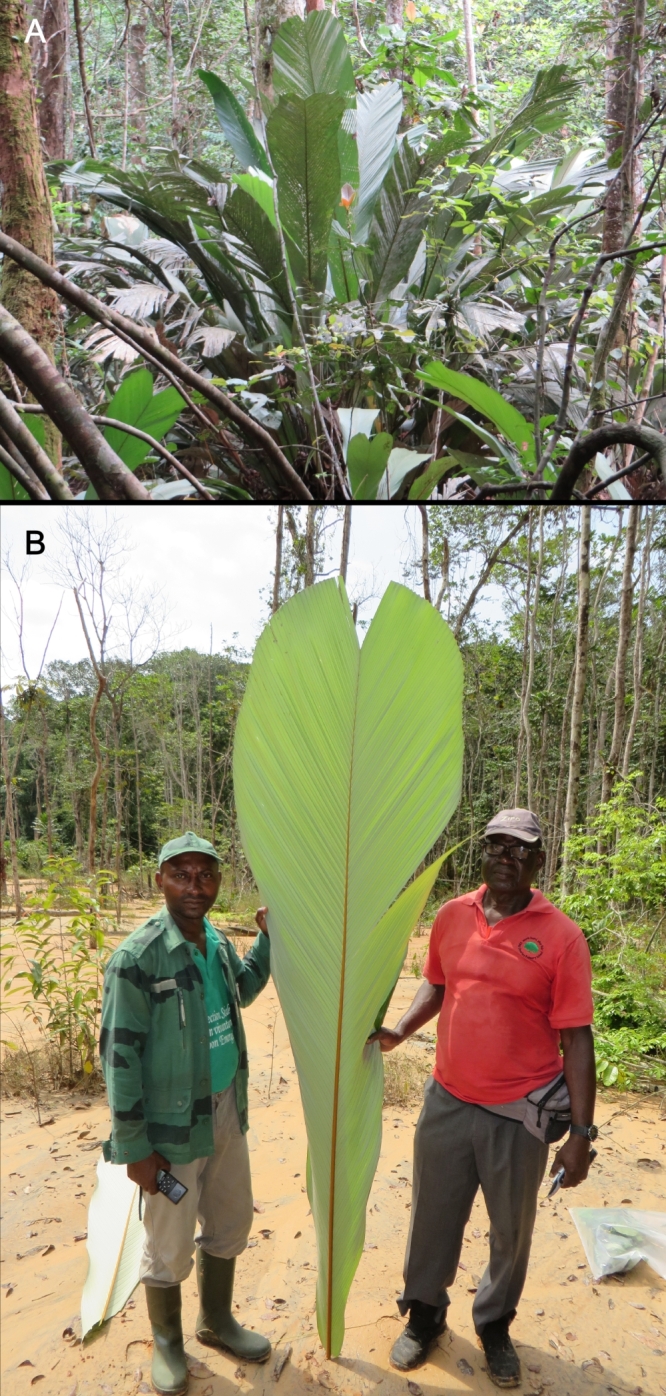
*Sclerosperma profizianum* . A) Habitat; B) Detail of the large bifid leaf. Photos: PH Bourobou Bourobou.

Sclerosperma profizianum Valk. & Sunderl. Kew Bull. 63(1): 82. 2008. Type: The Democratic Republic of Congo, Bas-Congo, terr. Madimba, Kisantu, 1913, Gillet 279a (WAG) (Fig. 3).

#### Materials

- **Type status:** Other material. **Occurrence:** recordNumber: P.H. Bourobou 1﻿748; recordedBy: Thomas Couvreur; **Taxon:** taxonID: urn:lsid:ipni.org:names:77090324-1; scientificName: Sclerosperma profizianum; kingdom: Plantae; class: Magnoliopsida ; order: Arecales; family: Arecaceae; genus: Sclerosperma; specificEpithet: profizianum; scientificNameAuthorship: Valk. and Sunderl.; **Location:** continent: Africa; country: Gabon; stateProvince: Ndindi; locality: Ndindi, Layon MPNM 11-26; locationRemarks: label transliteration: "﻿﻿Gabon, Ndindi, Layon MPNM 11-26, -3.857093, S 11.102234 E]"; verbatimCoordinates: 3° 51' 25.5348'' S; 11° 6' 8.0424'' E; verbatimLatitude: -3° 51' 25.5348''; verbatimLongitude: 11° 6' 8.0424'; decimalLatitude: -3.857093; decimalLongitude: 11.102234; geodeticDatum: WGS84; georeferenceProtocol: GPS; **Identification:** identifiedBy: Yves Issembe; dateIdentified: 2016; **Event:** eventDate: 2015-02-21; year: 2015; month: 2; day: 21; **Record Level:** language: french; collectionCode: LBV; basisOfRecord: PreservedSpecimen

#### Distribution

**Figure 4.**
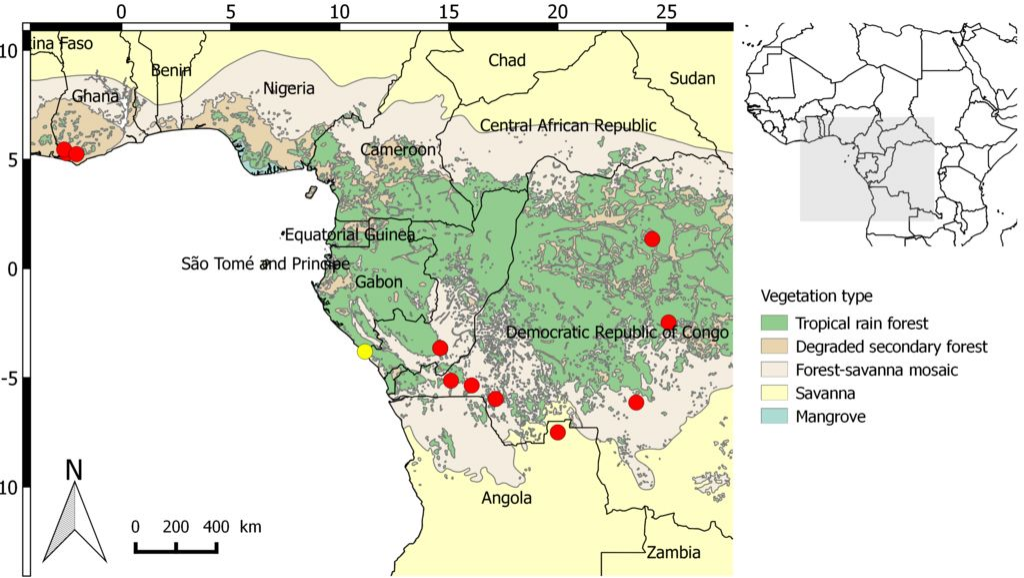
Updated distribution map of *Sclerosperma profizianum*. Yellow dots: New collection published here. Red dots: Distribution data from the RAINBIO dataset.

Documented occurrences: disjunct distribution between Ghana, Angola, the Republic of Congo and the Democratic Republic of Congo. (van Valkenburg et al. 2008) (Fig. 4​).

## Identification Keys

### Key to the species of *Eremospatha* in Gabon

**Table:** 

1	Leaflets unequally distributed, grouped by 2 or 3	*E. quiquecostulata*
–	Leaflets regularly distributed, not grouped by 2 or 3	2
2	Knee (swelling) clearly present on the stem under the petiole	3
–	Knee absent on the stem under the petiole	6
3	Leaflets rhomboid or trapezoid	*E. wendlandiana*
–	Leaflets not rhomboid or trapezoid	4
4	Leaflets less than 20 per side, cirrus spiny	5
–	Leaflets more than 20 per side, cirrus non spiny	*E. laurentii*
5	Leaflets obovate-elliptic, inflorescence glabrous	*E. hookeri*
–	Leaflets obovate to suborbicular; inflorescence profusely papillose to give brown velvety appearance	*E. cabrae*
6	Leaflet apex narrowly to broadly praemorse	7
–	Leaflet apex entire, terminating in a conspicuous apiculum	*E. cuspidata*
7	Cirrus spiny	*E. haullevilleana*
–	Cirrus non spiny	*E. macrocarpa*

Adapted from Sunderland (2012).

## Discussion

With the discovery of *S. profizianum* and *E. quiquecostulata*, Gabon is now home to 29 species of palms (nearly half the total number for continental Africa (Stauffer et al. 2014) making it one of the most important centers of diversity for this family in Africa (Blach-Overgaard et al. 2013).

*Eremospatha* is one of the four rattan genera found in Africa (Faye et al. 2014, Sunderland 2012, Sunderland 2007) which were recently revised by Sunderland (2012). To date *Eremospatha* contains a total of 12 species. *Eremospatha quiquecostulata* is easily distinguished by its leaflets that are inequidistant from one another and grouped by 2 or 3 (Fig. 1). To date, this species was known from Cameroon, with a single collection in Nigeria and one in Equatorial Guinea (Fig. 2). Here, we show that it is also present in Gabon, in the south and the north of the country. Gabon now has eight species of *Eremospatha*.

The genus *Sclerosperma*, with three species, was recently revised by van Valkenburg et al. (2008) in which a new species was published: *S. profizianum*. This species is characterized by having large bifid leaves (Fig. 3), in contrast to the two other species (*S. mannii* H. Wendl. and *S. walkeri* A. Chev.) with numerous pinnately composed leaves. This species was indicated to have a disjunct distribution between Ghana, Angola, the Republic of Congo and the Democratic Republic of Congo (Fig. 4). The collection of this species confirms the presence of all three *Sclerosperma* species in Gabon. Moreover, the Haute-Banio department is not well botanically explored with less than 100 botanical specimens found in the Herbier National du Gabon and Naturalis Biodiversity Centre, Leiden (Wieringa and Sosef 2011; Dauby et al. 2016). Potentially new species records for Gabon could be found there with further exploration. In terms of habitat, *Sclerosperma profizianum* was found in humid areas, in valley bottoms, periodically inundated forests or swamps along small rivers.

## Supplementary Material

XML Treatment for Eremospatha quiquecostulata

XML Treatment for Sclerosperma profizianum
